# Towards precision medicine in diabetes: pharmacogenomic approaches for MODY and type 2 diabetes with insights from GLIS3 gene variants

**DOI:** 10.3389/fendo.2026.1871527

**Published:** 2026-10-15

**Authors:** Abhisheka Lenin, Jasmin Sajini Rajayan

**Affiliations:** Sri Ramachandra Faculty of Pharmacy, Sri Ramachandra Institute of Higher Education and Research, Chennai, India

**Keywords:** GLIS3, Indian population, maturity-onset diabetes of the young, pharmacogenomics, precision medicine, type 2 diabetes, artificial intelligence in diabetes prediction, health equity and well being

## Abstract

**Background:**

Diabetes mellitus encompasses a heterogeneous group of metabolic disorders with a significant genetic basis that current standardized treatment protocols fail to adequately address. Type 2 diabetes in particular is driven by genetic factors that operate independently of lifestyle, with a substantial proportion of patients in South Asian populations developing the disease despite normal body weight and healthy habits. India presents a uniquely important context for pharmacogenomic research given its extraordinary population stratification across tribal, linguistic, religious, and geographic groups creating a genetic landscape that Western genomic data cannot adequately represent.

**Objective:**

This review evaluates pharmacogenomic evidence across monogenic and polygenic diabetes subtypes, presents Indian clinical case studies demonstrating the consequences of genomic misclassification, and positions GLIS3 as an underexplored regulatory gene with cross-disease relevance spanning neonatal diabetes, Type 1, and Type 2 diabetes. It further examines the potential for expanded clinical implementation of genotype-guided therapy, particularly in genetically diverse populations.

**Key insights:**

GLIS3 encodes a zinc finger transcription factor that governs beta-cell development, insulin gene transcription, and endoplasmic reticulum stress protection by functioning as a master regulator of beta-cell survival rather than acting through a single pathway. Its association across multiple diabetes subtypes, combined with emerging evidence of population-specific variants in Indian cohorts, positions it as a compelling future pharmacogenomic target. However, no GLIS3-directed clinical testing or therapy is currently available, and its pharmacogenomic utility remains hypothetical pending prospective validation. Indian pharmacogenomic data further reveal 76 known variants affecting antidiabetic drug response, with higher frequencies of poor metformin response alleles compared to global population findings with immediate clinical implications for one of the world’s highest diabetes-burden nations.

**Conclusion:**

Realizing the potential of precision medicine in diabetes requires population-specific genomic research, integration of pharmacogenomic testing into national health schemes such as Ayushman Bharat, development of AI-driven clinical decision tools, and policy frameworks guided by bodies such as ICMR. Genotype-guided therapy represents an emerging clinical priority with the potential to improve outcomes in genetically diverse populations where standardized protocols may be suboptimal.

## 1 Introduction

Diabetes mellitus is one of the most genetically complex diseases of our time, yet it continues to be managed through standardized treatment protocols that do not account for individual genetic differences. Published reports from Indian clinical centers document patients with monogenic diabetes who were treated inappropriately for years before a correct genetic diagnosis finally changed their management. These are not isolated incidents. They reflect a gap that exists across clinical settings, between what genomic science already knows and what actually happens when a patient walks into a clinic. This review is written to address that gap directly, with a particular focus on the Indian population and the underexplored regulatory gene GLIS3.

Diabetes mellitus is one of the most common and most misunderstood diseases in the world, affecting hundreds of millions of people globally with numbers still rising (1). Most people, including many clinicians picture diabetes as a disease of the overweight, the sedentary, the person who ate too much sugar for too long. While lifestyle factors do play a role, that picture is dangerously incomplete. A significant number of people develop diabetes despite having a healthy weight, a good diet, and an active lifestyle. Why? Because in many cases, the real driver is not what they ate or how much they exercised. It is their genes, and genes are not something anyone can control (2).

This represents a clinically significant observation that modern diabetes care has been slow to fully incorporate. Current treatment systems rely on standardized guidelines, doses, and drugs that assume population homogeneity. However, interpatient genetic heterogeneity limits the universal applicability of such standardized protocols. Two people can walk into the same clinic with the same HbA1c, the same BMI, and the same symptoms have completely different genetic profiles driving their disease. Giving them the same treatment is not precision medicine. This approach may lead to suboptimal treatment decisions.

Pharmacogenomics offers a potential solution to this problem. It is increasingly recognized as a clinically relevant field with significant potential. It changes the fundamental question from “what drug works for most people?” to “what drug works for this person, based on their genetic makeup?” (3). In monogenic diabetes, particularly in MODY subtypes, this revolution has already arrived. A single genetic test can move a patient from lifelong insulin injections to a simple oral tablet or in some cases, no medication at all. The evidence is clear. The benefit is real. The only thing missing is wider implementation.

Now the field is looking deeper toward genes that do not just influence drug metabolism but actually govern how the pancreatic beta-cell develops, functions, and survives under stress. Among these, one gene stands out for a very specific reason. Not because it is newly discovered, but because despite being studied for over a decade, its true potential has never been fully explored. That gene is GLIS3, a known player with unknown potential.

GLIS3 is a zinc finger transcription factor that plays a critical role in beta-cell differentiation, insulin gene expression, and protection against endoplasmic reticulum stress (4). What makes it remarkable is the range of its associations from severe neonatal diabetes caused by complete loss of function, to common Type 1 and Type 2 diabetes driven by subtle everyday variants. No other gene connects these dots quite the same way. That connection is exactly why GLIS3 deserves to be at the center of this conversation.

This review is based on the premise that genotype-guided treatment in diabetes represents an emerging clinical priority supported by expanding pharmacogenomic evidence. It is not written just to summarize existing literature, it is written to make an argument. An argument for change, in how we diagnose, how we treat, and how we think about diabetes especially in populations like India, where genetic diversity is unlike anywhere else in the world, and where the gap between what the science says and what actually happens in the clinic is still far too wide (5).

## 2 Literature search and selection (narrative review methodology)

This is a narrative review synthesizing published pharmacogenomic evidence relevant to diabetes mellitus. No systematic review protocol was registered, and no formal meta-analysis was conducted.

### 2.1 Databases and search strategy

A comprehensive search was conducted using PubMed, Google Scholar, and Scopus databases. The search was not restricted by date but prioritized studies published between January 2003 and May 2026, with particular emphasis on literature from 2020 onwards to ensure currency of evidence. Search terms included combinations of the following: “pharmacogenomics,” “diabetes mellitus,” “MODY,” “GLIS3,” “precision medicine,” “maturity-onset diabetes of the young,” “SLC22A1,” “PPARG,” “metformin pharmacogenetics,” “beta-cell dysfunction,” “Indian population genomics,” and “polygenic risk scores.” Additional searches were conducted specifically for GLIS3-related studies using terms including “GLIS3 neonatal diabetes,” “GLIS3 Type 1 diabetes,” “GLIS3 Type 2 diabetes,” “GLIS3 beta-cell,” and “GLIS3 South Asian populations.”

### 2.2 Inclusion criteria

Studies were included if they (i) reported original pharmacogenomic data in diabetes; (ii) reviewed gene-drug interactions relevant to antidiabetic therapy; (iii) described clinical outcomes in monogenic or polygenic diabetes in relation to genetic variants; or (iv) provided population-specific genomic data with relevance to Indian or South Asian cohorts. Case reports and cohort studies from Indian clinical centers were specifically sought to support the translational perspective of this review.

### 2.3 Exclusion criteria

Non-peer-reviewed sources (except government policy documents and established clinical guidelines), non-English publications, studies unrelated to endocrine pharmacogenomics, and purely bibliometric or scientometric analyses were excluded.

### 2.4 Screening and selection process

Titles and abstracts were screened for relevance, followed by full-text review of potentially eligible articles. Reference lists of included articles were manually screened for additional relevant publications. Evidence was selected based on clinical relevance, methodological quality, and recency, with priority given to studies reporting direct gene-drug interactions and population-specific pharmacogenomic data.

## 3 Precision medicine and pharmacogenomics in diabetes: a necessary revolution

Precision medicine, at its core, means treating every patient as an individual. Not just based on their symptoms or their test results, but based on who they actually are. Their weight, their lifestyle, their cultural background, their ethnicity, their family history, their mental and social health, and most importantly, their genetic makeup. In clinical practice, consultations often focus on glycemic levels and standard first-line therapy such as metformin.

“It should begin a deeper conversation where one that asks why this particular person has this particular disease, and what treatment will actually work for this particular body (6).

This is not merely an idealistic concept. It is increasingly being recognized as a clinical necessity.

Pharmacogenomics is the scientific foundation that makes this kind of individualized care possible. While it is often discussed as a future concept, the reality is that this revolution has already begun quietly, unevenly, but unmistakably. In India specifically, national genome projects are actively building population-specific databases of diabetes-related pharmacogenetic variants. Some tertiary institutions including AIIMS, Manipal, and CMC Vellore have begun experimenting with pharmacogenomic-informed prescribing, particularly in monogenic diabetes. Policy interest is growing alongside India’s rising diabetes burden, with precision medicine initiatives beginning to enter mainstream health discussions (7). We are not at the finish line, but we are well past the starting point.

The clearest proof that pharmacogenomics works comes from monogenic diabetes, particularly MODY. Here, the genetic logic is straightforward. One gene, one mutation, one clear treatment direction. A patient with an HNF1A mutation responds extraordinarily well to low-dose sulfonylureas. A patient with a GCK mutation may need no medication at all. The genotype tells the clinician exactly what to do, and the clinical evidence confirms it works (**Table 1**) (8). This is precision medicine in its purest form.

**Table 1:** Evidence grading of key pharmacogenomic targets in diabetes.

Gene	Drug/therapy	Mechanism	Evidence level	Population data	Clinical use
Monogenic diabetes — MODY					
HNF1A	Sulfonylureas (low dose)	β-cell transcription factor; sulfonylurea receptor hypersensitivity	High	European, Indian (Delhi cohort)	Routine clinical use
HNF4A	Sulfonylureas	Nuclear receptor; progressive β-cell failure with sulfonylurea sensitivity	High	European populations	Routine clinical use
GCK	No pharmacotherapy required	Glucokinase sensor; stable mild hyperglycemia	High	Global; Indian cohorts	Lifestyle only; no drugs
KCNJ11/ABCC8	Sulfonylureas (oral)	ATP-sensitive K^+^ channel; activating mutations allow switch from insulin	High	Global; Indian ABCC8 variants (South India cohort)	Routine—neonatal and MODY
HNF1B	Insulin (early initiation)	Pancreatic hypoplasia; reduced β-cell mass; sulfonylurea resistance	High	European; limited Indian data	Insulin required; multidisciplinary care
Type 2 diabetes — Polygenic					
SLC22A1	Metformin	OCT1 transporter; reduced hepatic metformin uptake with loss-of-function variants	Moderate	European; Indians show higher frequency of reduced-function alleles	Not routine; clinical caution advised
PPARG	Thiazolidinediones (TZDs)	Nuclear receptor; Pro12Ala variant alters insulin sensitivity	Moderate	European and Asian cohorts; inconsistent replication	Not routine; research setting
KCNJ11/ABCC8 (T2D)	Sulfonylureas	K^+^ channel variants modestly affect sulfonylurea sensitivity in polygenic T2D	Moderate	European; limited multi-ethnic data	Not routine in T2D
ATM	Metformin	DNA damage response; rs11212617 variant associated with metformin glycemic response	Moderate	European GWAS; limited validation	Not routine; investigational
Emerging target — GLIS3					
GLIS3	GLP-1 agonists/DPP-4 inhibitors (hypothetical)	Zinc finger transcription factor; regulates β-cell development, insulin transcription, ER stress protection	Emerging	Global GWAS; South Asian variant data (INSPIRED study, 2023); Indian-specific variants identified	Not yet clinical; potential biomarker and future therapeutic target

Evidence levels were assigned based on the following criteria: High = supported by randomized controlled trials, clinical practice guidelines, or replicated prospective cohort studies with consistent outcomes; Moderate = supported by well-powered observational studies, genome-wide association studies with independent replication, or consistent case-control data; Limited = supported by small cohorts, single-center studies, inconsistent replication, or indirect evidence; Emerging = supported by preclinical data, biological plausibility, hypothesis-generating studies, or expert consensus without prospective clinical validation.

Type 2 diabetes is a different and far more complicated story. It is not driven by one gene but by hundreds of common variants, each contributing a small effect, all of them interacting with lifestyle, environment, and each other. This polygenic complexity is exactly why no single genetic marker has yet been identified that reliably predicts drug response across all T2D patients (9). The challenge is not a lack of evidence it is a lack of clarity in a very noisy genetic signal (**Table 2**).

**Table 2:** Comparative overview of pharmacogenomic characteristics in monogenic versus polygenic diabetes with reference to the Indian population context and GLIS3 relevance.

Feature	Monogenic diabetes — MODY	Type 2 diabetes — polygenic
Genetic architecture	Single pathogenic gene variant; complete penetrance	Hundreds of common variants; small individual effects
Heritability	Very high; autosomal dominant inheritance	Moderate; significant environmental interaction
Typical age of onset	Before 35 years; often adolescence or early adulthood	Usually after 40 years; increasingly younger in South Asians
Body habitus	Usually lean or normal weight	Often overweight or obese; but not always in South Asians
Pharmacogenomic clarity	High; genotype directly predicts drug response	Low to moderate; polygenic effects confound prediction
Clinical utility of genetic testing	Established and routine in specialist centers	Limited; not yet routine; research setting only
Treatment implication	Genotype-specific: sulfonylurea, no medication, or insulin based on subtype	Standard guidelines with emerging pharmacogenomic refinement
Misdiagnosis risk	High; frequently misclassified as T1D or T2D	Low; clinical presentation is typical
Indian-specific data	Novel variants in RFX6, WFS1, AKT2, NKX6–1 identified; digenic mutations reported at CMC Vellore	Higher frequency of SLC22A1 reduced-function alleles; 76 pharmacogenetic variants identified in Indian populations
GLIS3 relevance	Shared β-cell transcription network; HNF1A, PDX1, NKX6–1 overlap with GLIS3 pathway	Common GLIS3 variants increase T2D risk; ER stress pathway relevant to β-cell loss in T2D

Yet even within this complexity, important pharmacogenomic patterns are emerging. One of the most clinically significant and most underappreciated involves metformin, the global first-line therapy for Type 2 diabetes. Metformin works by entering liver cells through a transporter protein called OCT1, encoded by the gene SLC22A1. In populations where reduced-function variants of this gene are common, metformin simply cannot get where it needs to go efficiently, and its glucose-lowering effect is diminished (10).

This matters enormously for India. A comprehensive pharmacogenomic analysis of Indian populations has catalogued 76 variants affecting non-insulin antidiabetic drug responses, including reduced-function alleles in SLC22A1 that impair metformin hepatic uptake (11). These reduced-function alleles occur at higher frequencies in Indian cohorts compared to European populations, suggesting population-specific differences in metformin transport efficiency (12) (11). In practical terms, this means that a drug given as standard first-line therapy to hundreds of millions of people may be working less effectively in a large proportion of Indian patients. Not because of anything they did wrong, but because of genetic variations in their drug transport system that no standard clinical assessment would ever detect.

This illustrates why pharmacogenomics represents a clinically relevant advancement. It distinguishes between prescribing a drug with demonstrated efficacy in a specific genetic context and prescribing a drug that may yield suboptimal glycemic control in genetically mismatched patients.

At the center of emerging pharmacogenomic research stands a gene whose potential has been consistently underestimated. GLIS3 is not new to science, it has been studied in the context of neonatal diabetes and thyroid development for years. But unlike genes such as KCNJ11 or ABCC8, which affect insulin release through a single ion channel mechanism, or SLC22A1, which affects only metformin transport, GLIS3 operates at a fundamentally different level. It is a master regulator that controls beta-cell development, governs insulin gene transcription, and protects beta cells from the endoplasmic reticulum stress that drives cell failure in chronic hyperglycemia (13). Its associations span the full disease spectrum, from rare neonatal diabetes to common Type 1 and Type 2 disease, connecting these different forms through a shared pathway of beta-cell health and resilience (**Figure 1**).

**Figure 1:**
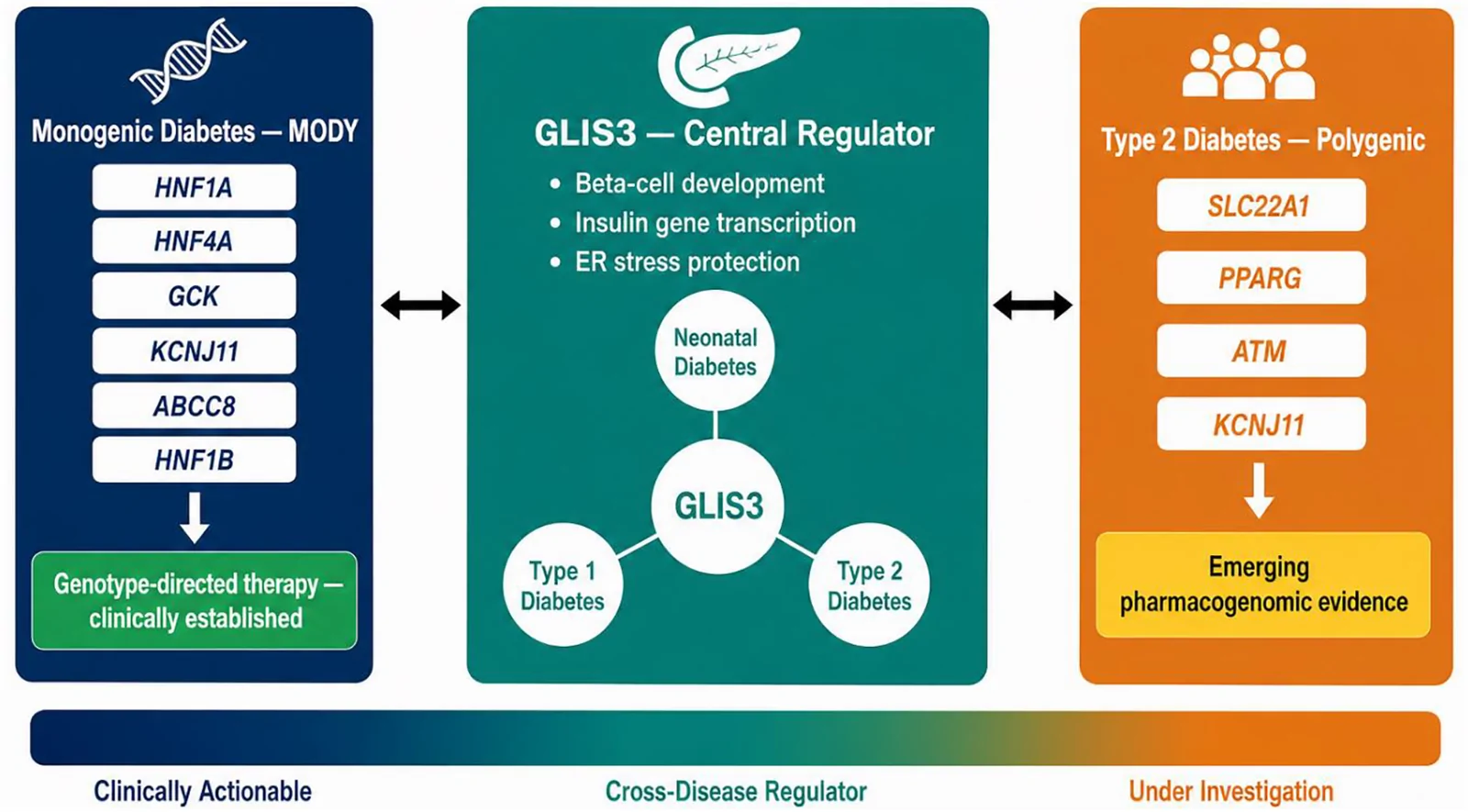
Unified pharmacogenomic framework in diabetes. The spectrum ranges from clinically established genotype-guided therapy in monogenic diabetes to emerging evidence in Type 2 diabetes, with GLIS3 positioned as a central regulatory factor connecting both disease forms through shared beta-cell biology. Figure created by the authors.

Understanding GLIS3 is not just academically interesting, it may be the key to developing therapies that work upstream by protecting the beta cell before it fails, rather than compensating for failure after it has already occurred.

## 4 Global and translational perspectives on pharmacogenomics in diabetes

Pharmacogenomics research has a fundamental problem. The vast majority of genomic data used to build drug-response guidelines comes from populations of European ancestry populations, whose diet, lifestyle, work culture, environmental exposure, and genetic background differ significantly from the rest of the world (11). Applying these guidelines universally is not just scientifically imprecise. In countries like India, it is clinically inappropriate.

Consider something as basic as diet and daily routine. Most Indian families follow food patterns that differ substantially from Western norms with heavier evening meals after long working hours, different macronutrient compositions, different cooking methods, and significant variation across regions, religions, and communities. Work stress levels, physical activity patterns, sleep cycles, and environmental exposures all differ too. These are not minor lifestyle details. These are factors that interact directly with genetic variants to shape how a drug.

However, the more profound issue runs deeper than lifestyle. India exhibits extraordinary genetic diversity shaped by thousands of years of population stratification across tribal and non-tribal communities, linguistic groups, and geographically distinct regions (14). This diversity has direct implications for pharmacogenomics: allele frequencies for drug-response variants can differ significantly between populations (11, 15). Ancient admixture events involving Iranian farmers, Central Asian steppe pastoralists, and indigenous Dravidian populations have created a genetic landscape of extraordinary complexity, that no other country quite replicates.

This complexity matters enormously for pharmacogenomics. Allele frequencies for drug-response variants can differ significantly between a tribal community in Odisha and an urban population in Chennai, between a Punjabi Sikh family and a Tamil Brahmin family. When clinicians prescribe metformin using dose guidelines developed from European cohorts to patients from Indian tribal or rural populations, the desired response may not occur, as pharmacogenomic studies demonstrate population-specific differences in SLC22A1 allele frequencies and metformin pharmacokinetics (11, 12).

Are Indian doctors aware of this? Awareness varies across clinical settings.

Awareness exists in academic and research institutions. Tertiary centers like AIIMS, CMC Vellore, and Manipal have begun integrating pharmacogenomic thinking into clinical practice, particularly for monogenic diabetes. But at the level of the general physician or the district hospital diabetologist who sees fifty patients a day, pharmacogenomics remains largely invisible. Guidelines are followed because guidelines exist, not because they were validated in Indian populations (16).

This gap between what the science knows and what the clinic does is not just an academic concern. It has real consequences for real patients who are given drugs that may work less effectively in their genetic background, who are misdiagnosed because diagnostic criteria were developed elsewhere, and who carry genetic variants that nobody thought to look for.

Closing this gap requires intervention at multiple levels simultaneously. Among the measures that have been proposed includes the national genomic databases, rural awareness campaigns, AI-driven clinical tools, public-private partnerships where one stands out as having the most immediate and scalable impact on ground-level patient care. It is the integration of pharmacogenomic testing into the Ayushman Bharat scheme.

Ayushman Bharat currently covers over 500 million Indians under its public health insurance framework (17). Integrating pharmacogenomic testing into this existing infrastructure represents a proposed policy direction rather than an implemented practice. If realized, such integration could improve access to precision medicine for underserved populations, though cost-effectiveness analyses and pilot implementation studies would be required before nationwide adoption. Rural campaigns and awareness drives are important, but awareness without access changes nothing. An ICMR national database is crucial for long-term research, but its benefits reach scientists before they reach patients. Ayushman Bharat integration reaches the patient directly, immediately, and at scale.

These observations highlight the need for policy-level interventions. India has the genetic diversity, the disease burden, the research infrastructure, and the public health framework to become a global leader in precision diabetes medicine. Realizing this potential will require policy commitment to connect existing research infrastructure with clinical implementation. Economic modelling suggests that pharmacogenomic integration into national health schemes could reduce misdiagnosis and long-term complication costs (17), though prospective health economic evaluations in the Indian context remain limited.

## 5 Maturity-onset diabetes of the young: lessons from Indian clinical experience

MODY represents the strongest existing proof that genotype-guided therapy works. In monogenic diabetes, a single genetic finding can completely redirect a patient’s treatment, not after years of trial and error, but immediately, precisely, and with evidence behind it. Yet despite this clarity, MODY remains one of the most consistently misdiagnosed conditions in clinical medicine, particularly in India, where genetic testing is still not part of routine diabetes workup (18).

The reasons are practical. Genetic testing requires time, infrastructure, specialized knowledge, and resources that most clinical settings do not have readily available. A standardized drug can be prescribed in minutes. A pharmacogenomic workup cannot. As a result, clinicians, who are already overburdened, resort to what is quickest, frequently at great expense to the patient (19).

Three case studies from Indian clinical settings illustrate exactly what this cost looks like in human terms.

### 5.1 Clinical case observations: delayed diagnosis and treatment shift

A 16-year-old lean girl arrived with diabetes and was treated as a Type 1 diabetic from childhood, with daily insulin injections, lifestyle restrictions, and all the psychological and physical burden that comes with it. Nothing about her phenotype screamed Type 1. She was lean. Her autoimmune markers were negative. Her C-peptide was preserved. She had a strong multigenerational family history of diabetes. But in the absence of genetic testing, the assumption held.

When genetic confirmation arrived, it identified an HNF1A mutation called MODY 3. The clinically significant finding was that she did not require insulin at all. She responded well to oral agents including vildagliptin and metformin. A decade of unnecessary injections, with all the associated treatment burden, anxiety, and cost, could have been avoided entirely if pharmacogenomic testing had been applied at the point of first diagnosis (20).

This is not a story about a medical mistake in the conventional sense. The doctors followed their training. They applied standard guidelines. The system failed her because, the system was not designed to look for what she actually had.

### 5.2 Insights from Indian genomic studies

A comprehensive genomic analysis of 289 individuals, including 152 clinically suspected MODY cases, produced findings that went far beyond confirming known MODY genes. Yes, HNF1A mutations were the most frequent at 7.2%, and ABCC8 mutations were identified in 3.3% of cases. However, the truly significant finding was the discovery of novel variants in genes which are not previously linked to MODY in global literature. That includes RFX6, WFS1, AKT2, and NKX6–1 (21).

This is the part that should concern every clinician and researcher working with Indian patients. Western diagnostic criteria for MODY were built on Western genomic data. When those criteria are applied to Indian populations, they are working with an incomplete map. Entirely new genetic pathways for diabetes were uncovered in this cohort pathways, that standard global guidelines would never have prompted anyone to look for. India’s genetic diversity is not just a demographic fact. It is a clinical reality with direct consequences for diagnosis and treatment.

### 5.3 Complex genetic patterns and treatment response

Eighty young-onset diabetes patients were sequenced using next-generation sequencing at CMC Vellore. Mutations were identified across multiple MODY-associated genes including HNF4A, HNF1A, GCK, PDX1, NEUROD1, and HNF1B. The discovery of digenic mutations, or patients having two defective genes at the same time, such as NEUROD1 and PDX1, was the most striking finding (22).

In these cases, standard therapies failed, not because the doctors chose the wrong drug, but because the underlying genetic architecture was so complex that no single-gene treatment assumption could hold. These patients were prescribed standard treatments based on conventional diagnostic criteria but did not respond, because their underlying genetic architecture was more complex than standard classifications assumed. Without genetic testing, the doctors were operating without sufficient genetic information.

Every one of these cases is different. Every one of these lives is equally important. Nevertheless, taken together, these cases indicate in India, MODY is not just underdiagnosed, it is actively mismanaged, at scale, because the tools to diagnose it correctly are not being used.

### 5.4 Implications for clinical practice

Even if the repair is difficult to implement, it is not complicated in concept. When a young, lean patient appears with diabetes, especially if there is a family history, preserved C-peptide, and no autoimmune markers, the clinical response should not be to instantly diagnose Type 1 or Type 2 diabetes and prescribe appropriately. It should be to pause, conduct a thorough investigation, and include genetic testing as part of the treatment plan.

Our role as clinicians and researchers is not simply to prescribe drugs. The goal is to save lives and reduce errors. Jumping to conclusions based on surface-level clinical signs when there are means to delve deeper is not efficient medicine, this may result in avoidable clinical consequences.

The Delhi girl did not need ten years of insulin. She needed one genetic test (23).

Expanding access to molecular diagnostics, training clinicians to recognize atypical diabetes presentations, and integrating genetic testing into standard diabetes workup protocols, particularly for young-onset cases are not optional upgrades to the system. They are the baseline standard that precision medicine demands.

## 6 Pharmacogenomics of type 2 diabetes and the GLIS3 spotlight

### 6.1 Why type 2 diabetes has no single pharmacogenomic answer

Type 2 diabetes does not follow simple genetic rules. Unlike monogenic diabetes where one faulty gene tells the whole story, T2D is the product of hundreds of common variants each contributing a small effect, all of them interacting simultaneously with lifestyle, diet, environment, and each other. No single gene dominates. No single variant reliably predicts how a patient will respond to a specific drug across different populations. This is the core reason why, despite decades of research and thousands of genome-wide association studies, T2D pharmacogenomics has not yet produced the kind of clean, clinically actionable guidelines that MODY has (24).

The genetic signal in T2D is real. But it is messy, overlapping, and context-dependent in ways that make it genuinely difficult to translate into prescribing decisions. A variant that predicts poor metformin response in a European cohort may behave differently in an Indian population because the surrounding genetic background, the environmental exposures, and the interacting lifestyle factors are all different (25). This is not a failure of pharmacogenomics as a concept. It is a reflection of biological complexity that the field is still learning to navigate.

Several variations have emerged as clinically significant. Reduced-function SLC22A1 variations impede hepatic OCT1-mediated metformin uptake, reducing the drug’s glucose-lowering efficacy, a result that is especially noteworthy in Indian communities where these variants occur more frequently (12). Polymorphisms in PPARG influence insulin sensitivity and thiazolidinedione response, though effect sizes remain modest and replication across diverse cohorts has been inconsistent (26). Variants in KCNJ11 and ABCC8 affect sulfonylurea sensitivity in T2D, though with far less predictive clarity than in monogenic diabetes (27). More recently, variants in the incretin signaling pathway have been associated with differential responses to GLP-1 receptor agonists and DPP-4 inhibitors, an area where research is still in its early stages (28).

Today’s honest assessment of T2D pharmacogenomics is that the biology is compelling, the relationships are real, but the therapeutic utility is restricted by small effect sizes, poor replication, and a persisting absence of large-scale prospective trials in various non-European populations. Progress is being made, although it is slower and more difficult than the MODY success story would suggest (29).

### 6.2 GLIS3 molecular biology and beta-cell mechanisms

GLIS3, GLI-Similar Zinc Finger Protein 3, is a member of the C2H2-type zinc finger protein family containing five zinc finger domains. It functions as both a transcriptional activator and repressor, binding specific DNA sequences to regulate gene expression across multiple tissues including the pancreas, thyroid, and kidneys (30). In the pancreatic beta cell, GLIS3 regulates biology through three principal mechanisms. First, it drives the differentiation of pancreatic progenitor cells into mature insulin-producing beta cells by regulating key developmental transcriptional programs. Second, it directly activates insulin gene transcription through binding to promoter and enhancer regions of the insulin locus, thereby maintaining adequate insulin biosynthesis (31). Third, it protects beta cells against endoplasmic reticulum stress by preserving protein-folding homeostasis, which is essential given the high biosynthetic demand of continuous insulin production (32, 33).

Loss of GLIS3 function disrupts beta-cell homeostasis through a defined pathophysiological cascade (**Figure 2**). Impaired insulin gene transcription reduces biosynthetic output and secretory capacity, while concomitant loss of ER stress protection permits accumulation of misfolded proinsulin within the endoplasmic reticulum. The resulting proteotoxic stress activates the unfolded protein response and downstream apoptotic signaling, including dysregulation of the BH3-only protein Bim, which ultimately drives beta-cell apoptosis (34). This mechanistic sequence directly links GLIS3 dysfunction to the progressive beta-cell loss observed in both Type 1 and Type 2 diabetes.

**Figure 2:**
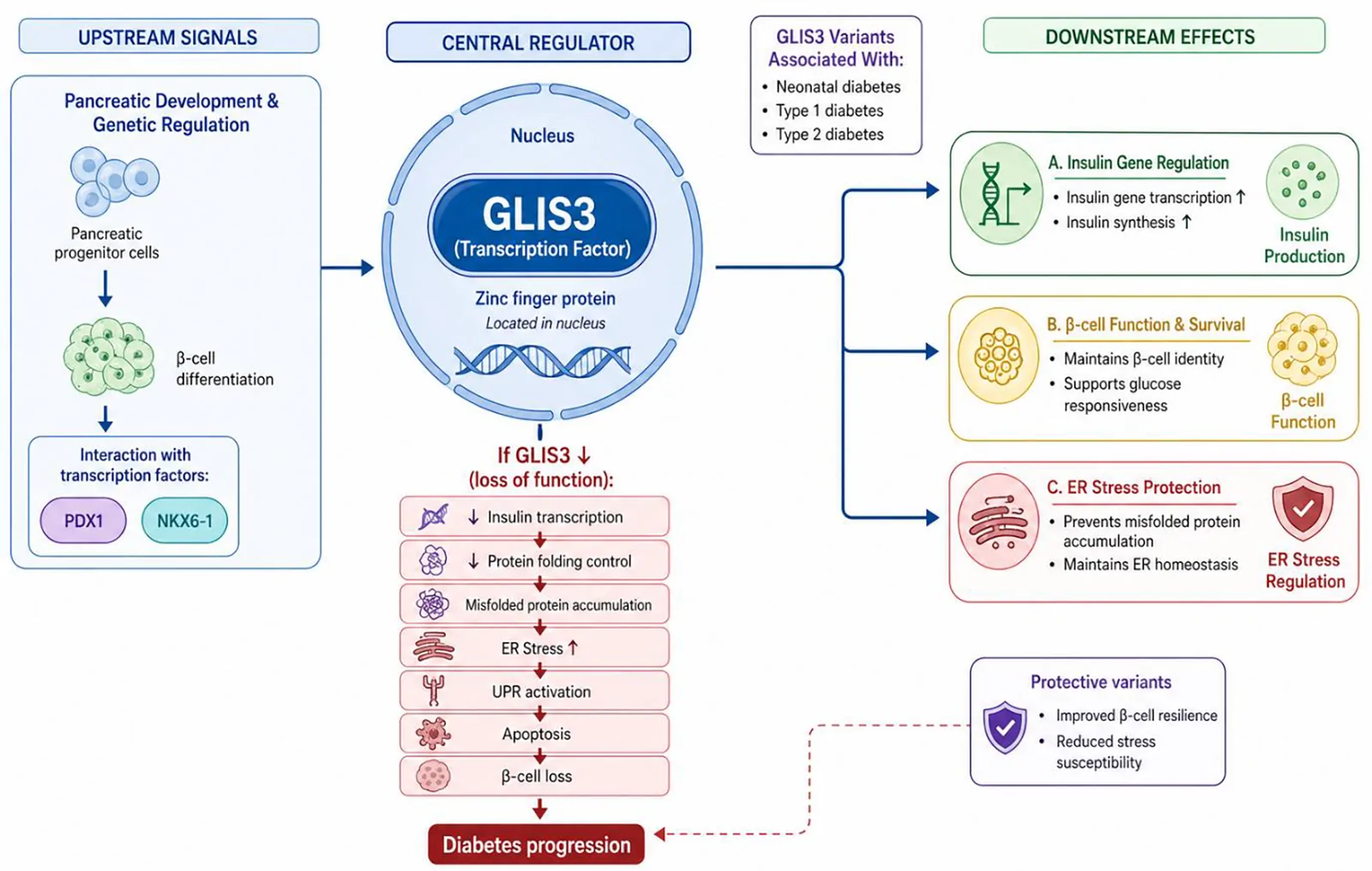
Role of GLIS3 in pancreatic β-cell regulation and diabetes pathophysiology. GLIS3 is a zinc finger transcription factor that regulates pancreatic β-cell development, insulin gene transcription, and cellular stress responses. It interacts with key transcription factors such as PDX1 and NKX6–1 to support β-cell differentiation and function. GLIS3 enhances insulin production and maintains β-cell identity while protecting against endoplasmic reticulum (ER) stress by preserving protein-folding homeostasis. Loss of GLIS3 function leads to impaired insulin transcription, accumulation of misfolded proteins, activation of ER stress pathways, and eventual β-cell apoptosis. Variants in GLIS3 have been associated with neonatal diabetes, Type 1 diabetes, and Type 2 diabetes, highlighting its role as a central regulator of β-cell survival across disease subtypes. This figure was generated with the assistance of artificial intelligence tools for visualization purposes; however, the conceptual design, scientific content, and interpretation are entirely the work of the authors.

The clinical spectrum of GLIS3 dysfunction reflects the severity of its mechanistic impact. Complete loss-of-function mutations cause neonatal diabetes with congenital hypothyroidism, confirming that GLIS3 is non-redundant for both beta-cell development and survival during the perinatal period (35). Partial loss-of-function or regulatory variants are associated with Type 1 and Type 2 diabetes susceptibility, consistent with a model in which diminished GLIS3 activity impairs insulin transcription and ER stress resilience, accelerating beta-cell apoptosis under metabolic or immune-mediated stress (34, 36).

### 6.3 GLIS3 as a pharmacogenomic hypothesis

The pharmacogenomic role of GLIS3 in guiding existing antidiabetic therapy remains a biologically plausible but clinically unvalidated hypothesis. GLIS3 loss-of-function variants may theoretically influence responses to therapies dependent on residual beta-cell function, including GLP-1 receptor agonists and DPP-4 inhibitors, given their reliance on a functional incretin-beta-cell axis. However, no clinical study has stratified drug response by GLIS3 genotype, and no pharmacogenomic guideline currently incorporates GLIS3 testing. The evidence presented herein supports GLIS3 as a susceptibility locus and mechanistic regulator, not as an established pharmacogenomic marker.

### 6.4 GLIS3 as a future therapeutic target

Beyond pharmacogenomics, GLIS3 represents a potential target for novel therapeutic development distinct from current drug-response stratification. Pharmacological augmentation of GLIS3 transcriptional activity or gene therapy approaches targeting beta-cell GLIS3 expression could theoretically protect against ER stress-mediated beta-cell failure (37). However, these possibilities remain at the preclinical stage. No GLIS3-targeted compound has entered clinical trials, and no gene therapy vector has been validated for this application. The development of small-molecule GLIS3 activators, beta-cell-specific delivery systems, and functional validation in human islet models represents a long-term research direction rather than a near-term clinical reality.

## 7 Clinical translation: when to use genetic testing and how to implement it

Translating pharmacogenomics into everyday clinical practice does not require reinventing how medicine works. It requires knowing when to look deeper than the surface.

In routine clinical care, a doctor evaluating a new diabetes patient will consider age, weight, symptoms, previous medication history, diet, and lifestyle before making a prescribing decision. First-line therapy is initiated, follow-up is scheduled, and in some cases imaging or additional tests are ordered to monitor progress. This is standard practice and it is not wrong. However, it has a critical blind spot which assumes the prescribed drug will work, without ever asking whether this particular patient’s genetic background supports that assumption.

The clearest signal that genetic testing is needed is treatment failure despite proper adherence. When a patient consistently takes their medication, follows dietary guidance, and makes lifestyle modifications but still exhibits no significant improvement in glycemic control, it is time to conduct a more in-depth assessment. At that point, continuing to adjust doses of a medicine that may never work for this patient’s genetic profile does not constitute perseverance. At that point, continuing to adjust doses of a drug that may never work for this patient’s genetic profile is not persistence, it is delay (38).

Specific clinical red flags that should prompt genetic workup include young age of onset particularly below 30 years, lean body habitus in a patient diagnosed with Type 2 diabetes, strong multigenerational family history of diabetes, negative autoimmune markers in a presumed Type 1 patient, preserved C-peptide suggesting residual beta-cell function, and unexpected or disproportionate response or non-response to standard therapy (39).

Ideally, genetic testing should be considered at the point of first diagnosis, particularly when these red flags are present. However, the practical reality of healthcare in India and other low and middle-income countries means that not every patient can afford advanced genetic workup at their first clinical visit. In these situations, a structured strategy is more realistic in-order to begin normal therapy, monitor response adequately, and escalate to genetic study if treatment fails or the clinical picture remains ambiguous (40).

This staged approach is not a compromise of precision medicine values. It is a pragmatic adaptation to resource-limited environments. What matters is that genetic testing eventually happen. Not that it happens last, after years of inappropriate treatment, but at the earliest clinically justified opportunity.

For this to become reality at scale in India, the government must play an active role. Schemes like Ayushman Bharat need to extend coverage to pharmacogenomic testing. Regional genomic testing centers need to be established and linked to district hospitals. Rural awareness campaigns need to reach communities where diabetes goes undetected for years. And medical education needs to prepare the next generation of clinicians to think genetically from day one of their clinical training (41).

Because at the end, optimizing clinical practice to reduce diagnostic errors and improve treatment outcomes remains a central goal of precision medicine implementation. Every year that passes without a correct genetic diagnosis for a MODY patient is another year of avoidable harm. That is not acceptable when the tools to prevent it exist.

## 8 Challenges and the path forward

The science of pharmacogenomics in diabetes is compelling. The evidence is growing. The benefits in monogenic diabetes are already proven. Nevertheless, the gap between what research shows and what actually happens in clinical practice remains substantial. Understanding why that gap exists and what it will genuinely take to close it is as important as any molecular finding this field has produced.

The first and most fundamental challenge is the diversity crisis in genomic data. Approximately 80% of all genome-wide association study participants to date are of European ancestry, despite this group representing only around 16% of the global population (42). For a country like India, which carries more genetic diversity within its borders than most continents, this is not a minor statistical imbalance. It means that the pharmacogenomic guidelines being applied to Indian patients were built on data that does not represent them. Allele frequencies differ. Gene-environment interactions differ. Drug response patterns differ Until large-scale genomic studies are conducted in South Asian, African, Middle Eastern, and Latin American populations, precision medicine will remain precise only for a minority of the world (5).

The second challenge is the modest effect size problem in Type 2 diabetes. Unlike MODY where one variant changes everything, T2D variants each contribute a small, incremental effect on drug response. Individually, most are not clinically actionable. Collectively, they paint a picture, but reading that picture requires polygenic risk scoring approaches and large prospective datasets that most healthcare systems are not yet equipped to use routinely (43). Many associations identified in initial studies have also failed to replicate consistently across different populations, partly because of differences in how outcomes are measured, how confounders are controlled, and how diverse the study cohorts are. This inconsistency has slowed clinical adoption and justifiably made clinicians cautious.

The third challenge is the knowledge gap at the clinical level. Pharmacogenomics is discussed in research institutions and academic medical centers. But most general physicians and diabetologists working in district hospitals and primary care settings were trained in an era before genomic medicine became clinically relevant. They follow standard guidelines because standard guidelines are what they have. Asking them to interpret a complex genetic report and translate it into a prescribing decision without support tools, without training, and without clear institutional protocols, is asking too much (44). The solution is not to blame clinicians. It is to build systems that make genomic information interpretable and actionable at the point of care.

The fourth challenge is infrastructure and cost. Genetic testing remains expensive and inaccessible in most low and middle-income countries. Laboratory capacity for next-generation sequencing is concentrated in a small number of tertiary institutions. Genetic counsellors are scarce. Reimbursement frameworks do not exist. And without policy intervention, market forces alone will not solve this, because the patients who need precision medicine most urgently are often the ones least able to pay for it (45).

Finally there is the ethical dimension. As genomic data collection scales up, questions of data privacy, informed consent, genetic discrimination in insurance and employment, and equitable access become increasingly urgent. A future where precision medicine exists but is available only to wealthy urban patients is not a better future, it is a more unequal one. These concerns must be built into the framework of pharmacogenomics implementation from the beginning, not addressed as an afterthought (46).

Recent large scale genomic studies have further confirmed the extent of heterogeneity across populations and the need for more inclusive research designs (47, 48). The genetic drivers of T2D pathophysiology identified in diverse cohorts are already revealing variants and pathways that European-centric studies missed entirely (49). Emerging data on GLIS3 variants specifically in South Asian populations is beginning to establish the cross-ethnic relevance of this gene as a pharmacogenomic target (36).

The path forward requires simultaneous progress on multiple fronts with more diverse research populations, standardized outcome measures for pharmacogenomic trials, clinical decision support tools embedded in electronic health records, updated medical education curricula, and national health policies that treat genetic testing as an essential service rather than a premium one. None of these is easy. But none is impossible either. And the cost of inaction, measured in misdiagnoses, inappropriate treatments, and preventable complications, grows every year.

## 9 Future directions: emerging priorities in precision diabetes medicine

The progress made in diabetes pharmacogenomics over the past two decades is real and meaningful. But progress is not the same as arrival. The most important work in this field has not yet been done and the directions it must take are becoming increasingly clear.

### 9.1 Artificial intelligence and machine learning in precision diabetes

Type 2 diabetes is too genetically complex for any single variant to carry clinical decision-making alone. Hundreds of common variants, each contributing a small effect, interact with environmental exposures, lifestyle factors, and each other in ways that no human mind can calculate simultaneously at the point of care. This is precisely the problem that artificial intelligence is designed to solve.

Machine learning models trained on large, diverse genomic datasets can identify patterns of gene-drug interaction that conventional statistical approaches miss. They can integrate genetic data with clinical parameters, laboratory values, medication history, and lifestyle information to generate personalized treatment recommendations in real time (47). In diabetes, AI-driven polygenic risk score techniques are already showing potential in stratifying patients based on their expected response to metformin, sulfonylureas, and GLP-1 receptor agonists, moving away from single-variant analysis and toward a systems-level perspective of medication responses.

For India, AI tools offer a particularly important opportunity. Given the extraordinary genetic diversity of the Indian population and the scale of the diabetes burden, manual interpretation of pharmacogenomic data by individual clinicians is not a scalable solution. AI-driven clinical decision support tools embedded directly into electronic health record systems could flag pharmacogenomically relevant findings automatically by alerting a prescribing doctor that a patient’s genotype suggests reduced metformin efficacy before the first prescription is written (48). This is not futuristic. The technology exists. What is needed is the will to implement it.

### 9.2 Multi-omics integration

Genomics alone tells only part of the story. Gene expression levels, protein abundance, metabolite profiles, and epigenetic modifications all influence how a patient responds to a drug and these factors vary across individuals, across time, and in response to treatment itself. Multi-omics approaches that combine genomic data with transcriptomic, proteomic, and metabolomic information are beginning to reveal drug-response mechanisms that single-omic studies cannot detect (49).

In the context of GLIS3, multi-omics integration is particularly promising. Single-cell transcriptomic studies have already begun to reveal how GLIS3 expression varies across beta-cell subpopulations and functional states. Combining this with proteomic data on the downstream targets of GLIS3 transcriptional activity, and metabolomic data on beta-cell energy metabolism under stress conditions, could map the full regulatory network that GLIS3 controls and identify specific nodes within that network that could be targeted therapeutically (36).

### 9.3 Polygenic risk scores in clinical practice

Polygenic risk scores aggregate the effects of thousands of genetic variants into a single predictive metric that represents one of the most practical near-term applications of genomics in T2D management. Rather than looking for one gene that predicts drug response, polygenic risk scores capture the cumulative genetic burden across the entire relevant genome (50).

In pharmacogenomics, this approach is beginning to be applied to predict metformin response, sulfonylurea sensitivity, and risk of drug-induced hypoglycemia. For Indian patients specifically, developing population-specific polygenic risk scores trained on Indian genomic data rather than applying European-derived scores is an urgent research priority. The genetic architecture of T2D in Indian populations is distinct enough that European polygenic risk scores show reduced predictive accuracy when applied across ethnicities (51).

### 9.4 GLIS3 as a potential therapeutic target

The most exciting future direction specific to this review is the development of GLIS3-targeted therapeutic strategies. Two approaches deserve particular attention.

First, GLIS3 variant status as a potential future biomarker. The hypothesis that patients with loss-of-function or reduced-function GLIS3 variants exhibit greater beta-cell vulnerability under metabolic stress requires validation in prospective cohorts. If confirmed, GLIS3 genotyping may inform stratified therapeutic approaches, including earlier use of beta-cell protective agents. However, no clinical trial has stratified antidiabetic therapy based on GLIS3 genotype, and this application remains speculative (52).

Second, pharmacological or genetic augmentation of GLIS3 activity. Small molecule compounds that enhance GLIS3 transcriptional activity, or gene therapy approaches that restore GLIS3 function in dysfunctional beta cells, represent a genuinely novel therapeutic direction. The goal would be upstream intervention protecting the beta cell before it fails, rather than compensating for failure after it has occurred. This approach has never been tested clinically, but the biological rationale is strong and the mechanistic groundwork is being laid (53).

### 9.5 Specific future research priorities for GLIS3

Realizing the clinical potential of GLIS3 in diabetes management will require coordinated research across multiple domains. Priority areas include: (i) functional validation of GLIS3 variants in human islet models and stem-cell-derived beta cells to confirm mechanistic effects on insulin transcription and ER stress resilience; (ii) large-scale South Asian cohort sequencing and pharmacogenomic association studies to establish population-specific GLIS3 allele frequencies and drug-response relationships; (iii) prospective genotype-stratified clinical trials of incretin-based therapies in patients with GLIS3 loss-of-function variants; (iv) development of beta-cell stress models to screen for small-molecule GLIS3 modulators; and (v) integration of GLIS3 variant data into AI-driven clinical decision support systems for diabetes management.

### 9.6 Toward a national framework for pharmacogenomics implementation

India has everything it needs to become a global leader in precision diabetes medicine with extraordinary genetic diversity, a massive patient population, world-class research institutions, and a government health infrastructure capable of nationwide reach. What is missing is a coordinated national strategy.

The roadmap for that strategy is clear. In the short term, integrate pharmacogenomic testing into Ayushman Bharat coverage, establish regional genomic testing centers linked to district hospitals, and begin training medical and pharmacy students in clinical genomics. In the near term, an ICMR-led national pharmacogenomic database representing of India’s full ethnic variety will be developed, as will AI-driven clinical decision support systems calibrated to Indian demographic data and public awareness campaigns in rural and urban settings. Long-term goals include developing India-specific clinical recommendations for pharmacogenomic testing in diabetes, forming public-private partnerships with biotech and pharmaceutical companies, and contributing Indian genomic data to global precision medicine initiatives (54).

This represents a feasible policy framework grounded in existing infrastructure and it is one that will only happen if researchers, clinicians, policymakers, and the government commit to it together.

### 9.7 Limitations

This review has several limitations that should be acknowledged. First, as a narrative review, it does not employ systematic review methodology or meta-analysis; therefore, quantitative effect estimates for pharmacogenomic associations could not be derived. Second, large-scale prospective pharmacogenomic validation studies in South Asian populations remain limited, and many of the population-specific claims discussed herein rely on smaller cohort studies or biological plausibility rather than replicated clinical trials. Third, no prospective GLIS3-directed clinical trials or genotype-stratified therapeutic studies have been conducted to date; consequently, all discussion of GLIS3 pharmacogenomic utility or therapeutic targeting remains hypothetical. Fourth, the translation of pharmacogenomic testing into national health schemes such as Ayushman Bharat is presented as a policy proposal rather than an implemented intervention, and cost-effectiveness analyses specific to the Indian context are not yet available. Finally, the generalization of findings from monogenic diabetes to polygenic Type 2 diabetes, particularly regarding GLIS3-mediated beta-cell mechanisms, requires cautious interpretation given the differing genetic architectures involved.

## 10 Conclusion

Diabetes is not one disease. It never was. It is a spectrum of conditions driven by different genes, different mechanisms, and different biological realities and it demands different treatments. The evidence reviewed indicates that continuing to treat every diabetes patient identically may represent suboptimal care, with potential consequences for long-term patient outcomes.

In monogenic diabetes, this is already proven. The Delhi girl who spent a decade on unnecessary insulin did not need more medication. She needed one genetic test. The South India cohort that revealed entirely new MODY variants invisible to Western diagnostic criteria did not need better drugs. It needed better tools. The CMC Vellore patients with digenic mutations who failed standard therapy had complex genetic architectures not captured by conventional diagnostic approaches. These are not isolated findings. They are warnings and they are being repeated every day in clinics across India and the world (55).

In Type 2 diabetes, the path is harder but the direction is clear. No single variant dominates. No single marker predicts everything. But the cumulative weight of pharmacogenomic evidence from SLC22A1 and metformin response, to PPARG and thiazolidinedione sensitivity, to emerging polygenic risk score that approaches towards a future where T2D prescribing is informed by genetic data rather than population averages (56).

At the center of that future stands GLIS3. Not because it is the most studied gene in diabetes. But because it may be the most important one we have not yet fully understood. It is the overlooked switch that decides whether a beta cell survives the stress of nonstop insulin production. It connects neonatal diabetes, Type 1, and Type 2 through a shared pathway of beta-cell health. It offers both a potential biomarker opportunity for identifying patients at risk of beta-cell failure and a future therapeutic opportunity for protecting beta cells upstream. Both applications remain at the preclinical and hypothesis-generating stage (34). The research community owes GLIS3 far more attention than it has received.

For India specifically, this review is a call to action. India has the genetic diversity, the disease burden, the scientific talent, and the public health infrastructure to lead the world in precision diabetes medicine. What it needs is the political commitment to connect these assets into a coordinated national strategy that integrates pharmacogenomic testing into Ayushman Bharat, builds an ICMR national genomic database, trains the next generation of clinicians in genomic medicine, and ensures that a patient in rural Tamil Nadu has the same access to genotype-guided therapy as a patient in a Delhi research hospital (57).

Medical science is advancing faster than health policy in most parts of the world. Bridging that gap requires more than data. It requires conviction that every patient deserves treatment designed for them specifically, not for a theoretical average patient.

Precision medicine in diabetes aims to optimize therapeutic outcomes through individualized, genetically informed care. The implementation of these strategies warrants serious policy and clinical consideration.
